# A Nationwide Study: Medical Students’ Perceptions of Plastic Surgery and Its Role in Career Choice in Saudi Arabia

**DOI:** 10.7759/cureus.73542

**Published:** 2024-11-12

**Authors:** Razan Omar Alsubhi, Isra'a Abdulaziz Alzahrani, Abdulah Bokhari, Rena Y Abualjamal, Jumanah Hamed Alqurashi, Rahaf Issa Almughamsi, Basem Alhusaini

**Affiliations:** 1 College of Medicine, Taibah University, Medina, SAU; 2 College of Medicine, Umm Al-Qura University, Makkah, SAU; 3 College of Medicine, King Saud Bin Abdulaziz University for Health Sciences, Jeddah, SAU; 4 College of Medicine, Imam Mohammad Ibn Saud Islamic University, Riyadh, SAU; 5 Department of Plastic and Reconstructive Surgery, King Fahad Genaral Hospital, Madinah, SAU

**Keywords:** career decision, medical students' perceptions, plastic surgery, plastic surgery training, saudi arabia

## Abstract

Background

Plastic surgery is a dynamic field within medicine, shaped by various factors that impact medical students' perceptions and career choices. This study aimed to assess medical students’ perceptions of plastic surgery as a specialty career and to examine factors influencing it, particularly understanding the impact of demographics, educational exposure, and media.

Methods

A cross-sectional study involved 777 medical students from diverse universities across Saudi Arabia. Students from both public and private medical colleges participated by completing an online questionnaire detailing their perceptions, exposure to plastic surgery, and career interests. Chi-square tests were used for the comparative analysis.

Results

The majority of participants were females, 64.2% (499 of 777), and only a small fraction expressed a strong interest in pursuing plastic surgery as a specialty, 10.9% (85 of 777), with social media and the internet emerging as influential sources of information. A majority of the students, 71.7% (557 of 777), acknowledged the impact of medical dramas on their career choice, while 42.3% (329 of 777) admitted to infrequently researching the topic online. Interestingly, while overall interest in plastic surgery was modest, preferences varied across clinical scenarios, with orthopedics and neurology/neurosurgery also receiving consideration. Academic performance, as measured by grade point average (GPA), did not significantly correlate with students' perceptions of plastic surgery. However, among female students, watching medical dramas was a significant influencing factor with a p-value of 0.001.

Conclusion

The findings highlight the complexity of factors influencing medical students' views on plastic surgery, stressing the need for focused educational efforts and mentorship initiatives. Early intervention to correct misconceptions and increase exposure to the specialty during medical training is crucial for cultivating interest and developing a knowledgeable workforce in plastic surgery.

## Introduction

Plastic surgery, which encompasses cosmetic and reconstructive procedures, is a multifaceted surgical specialty with diverse subspecialties such as microvascular surgery, hand surgery, and cranio-maxillofacial surgery. Despite its growing demand over the past two decades, there remains a significant shortage of adequately trained plastic surgeons. This discrepancy was predicted to be due to the rapid growth of the field but fixed number of residency training program seats [1]. According to the same study conducted in the United States, this trend is projected to worsen by 2025 [1]. Comparatively, Saudi Arabia suffers a similar shortage of plastic surgeons. This scarcity could also be attributed to the widespread lack of understanding of plastic surgery not only among the general public but also among medical students and practitioners in other fields [2]. 

In Saudi Arabia, which has over 30 medical colleges, the educational system primarily consists of seven years of medical education to qualify as a general physician, followed by 6 years of full-time training in plastic surgery and its branches to become a Saudi board-certified plastic surgeon. The first 7 years vary across universities in terms of the inclusion of plastic surgery in the curriculum, while the residency training program is unified across the country. Research suggests that exposure of medical students to this specialty during college can positively influence their perceptions of plastic surgery and its subspecialties. However, existing studies have focused on single institutions or regions and have not provided a nationwide perspective [2-5]. For instance, a study at King Faisal University in Al-Ahsa highlighted the need for increased awareness of plastic surgery, particularly hand surgery, among both students and interns [3]. Similarly, an investigation at King Abdulaziz University, Jeddah, revealed that although medical students had adequate knowledge of burn treatment, they had limited awareness of other plastic surgery subspecialties [4]. The evident lack of an inclusive study highlights the need for a nationwide study, as differences across regions and institutions can vary with variations in social norms and culture. A national perspective is crucial to facilitate policymaking and ensure that the diverse needs and challenges of the country are addressed.

Various factors other than the curriculum also contribute to misconceptions about plastic surgery among Saudi Arabian medical students. These factors include insufficient media coverage, negative societal perceptions of plastic surgeons, and the conservative nature of the country’s culture. Hence, addressing these factors is crucial to fostering a better understanding of plastic surgery and promoting interest in this specialty among medical students nationwide.

Considering its status as one of the most competitive fields in medicine, the perception of plastic surgery among medical students is of significant concern. Developing an early understanding of this specialty is crucial for students aiming to secure placements in this highly competitive program. A recent cross-sectional study conducted in the western region in 2021 emphasized how misconceptions about plastic surgery directly impact medical students’ likelihood of pursuing it as a career in the future [6]. Furthermore, medical students' misconceptions of plastic surgery can yield further social stigma and widen the gap in societal awareness.

Hence, assessing medical students’ perceptions of plastic surgery is paramount. Detecting and rectifying any misconceptions early on can mitigate future issues, such as erroneous referrals and consultations when these students become practicing physicians. In addition, gauging the extent of misperceptions can inform medical colleges to revise their curriculum so that courses on plastic surgery are included. Without such highlights of the widespread misconceptions, the predicted shortage in the plastic surgery field will not be resolved. Furthermore, remediation of the issue at hand will positively impact patient satisfaction and the quality of care provided.

Although several studies have examined the misconceptions pertaining to plastic surgery among medical students in specific regions and institutions, nationwide studies addressing this issue are lacking. Therefore, this gap should be bridged by evaluating the perceptions of plastic surgery among medical students across the country and their impact on career choice. This study aimed to assess the perception of plastic surgery among medical students in Saudi Arabia, explore the factors influencing medical students’ consideration of this specialty as a career choice, and compare these factors based on demographic and medical-related characteristics of the students. 

## Materials and methods

This cross-sectional study was conducted between September 2023 and December 2023 in Saudi Arabia to capture medical students’ perceptions of plastic surgery and their influence on career choices. Medical students enrolled in public and private universities across Saudi Arabia were recruited for this study. Interns, residents, and physicians of all medical specialties were excluded from the study sample.

**Figure 1:**
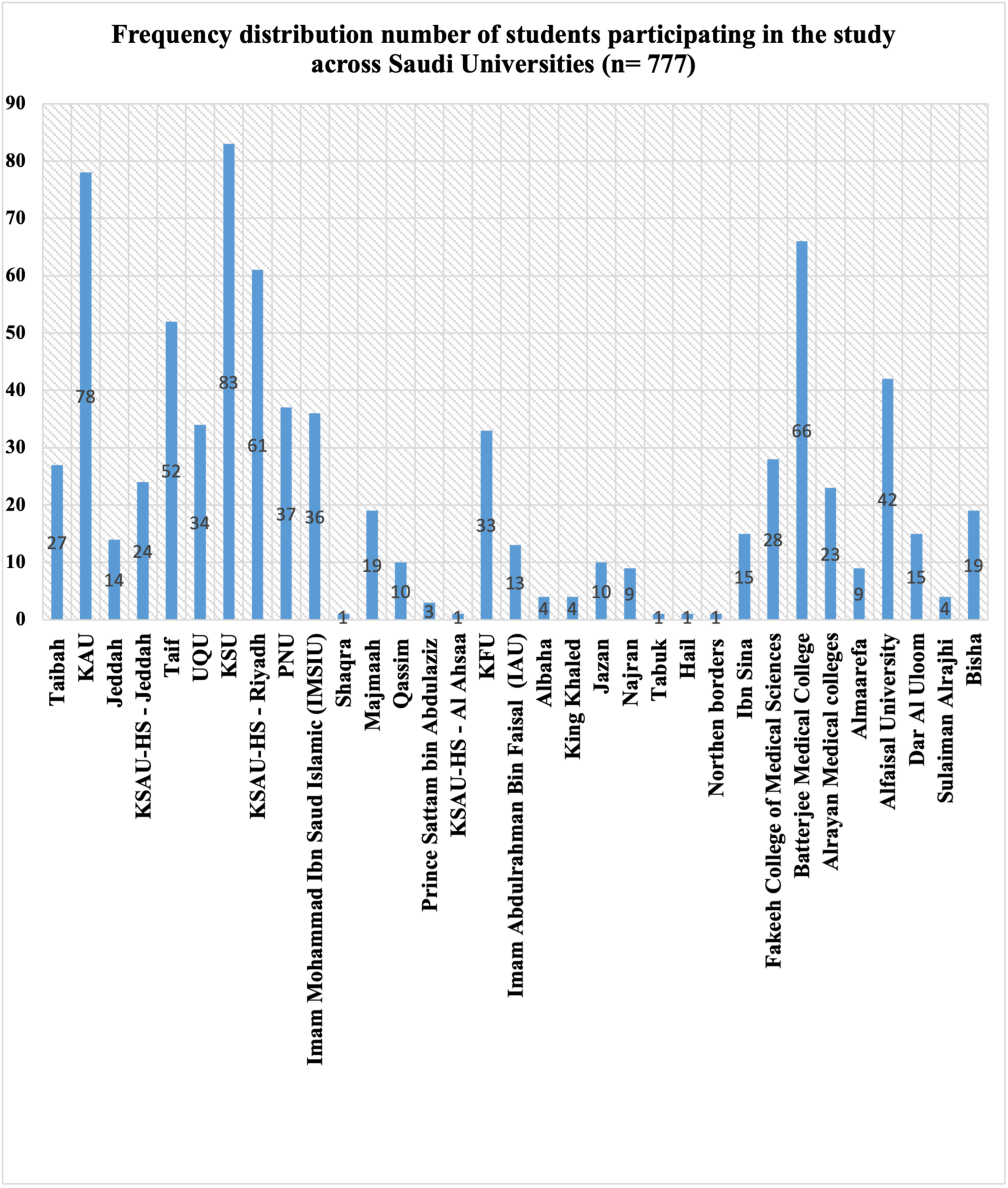
Bar chart showing the number of students participating in the study from each studied university.

Convenience sampling was used to recruit participants from different universities in KSA via online platforms. A minimum of 5% of each university's students responded to our questionnaire; names of the universities included in this study and the number of medical students participating from each studied university are provided in Figure 1.

Data were collected using a self-administered online questionnaire, which was distributed by the data collectors from each university via WhatsApp groups and social media. The questionnaire was adapted and modified from previously published articles [3,6] and was prepared after an extensive literature review of factors influencing the choice of plastic surgery as a specialty among medical students. A pilot study was conducted on a representative sample to validate the questionnaire before full-scale distribution. A pilot study was conducted on a representative sample to validate the questionnaire before the full-scale distribution. Based on its results minor changes and technical modifications were implemented to enhance the clarity of the questionnaire

The questionnaire included demographic data of the examined students, such as age (<20, 20-25, and >25 years), sex (male and female), marital status (single, married, divorced, and widowed), monthly family income (<5000, 5000 to <10000, 10000-15000, and >15000 SR), educational year (2nd, 3rd, 4th, 5th, and 6th year), and academic performance represented as grade point average (GPA; <2.75, 2.75-3.74, 3.75-4.4, and 4.5-5). Furthermore, the questionnaire included data related to medical students’ perceptions of plastic surgery, factors influencing plastic surgery as a career choice, exposure to plastic surgery during medical education, and personal interest in the specialty. The questionnaire was validated by a panel of experts in plastic surgery and medical education to ensure the relevance and clarity of the items.

The collected data were recorded and analyzed using the Statistical Package for the Social Sciences (version 22.0; SPSS Inc., Chicago, IL). Frequency distributions and percentages were used to summarize the demographic characteristics and responses to questionnaire items. Chi-square tests were employed to compare the factors affecting the choice of plastic surgery as a specialty and the students’ demographic and educational factors. A p-value of ≤0.05 was considered to be statistically significant.

Ethical approval for the study was obtained from the ethics committee before data collection. Moreover, informed consent was obtained from all participants before they completed the questionnaire, emphasizing voluntary participation and confidentiality of responses. In addition, adequate measures were taken to ensure data security and anonymity throughout the study process.

## Results

The demographic profile of the study participants is outlined in Table 1. Of the 777 students surveyed, 64.2% were women and 35.8% were men. The predominant age group was 20-25 years, which comprised 88.8% of the sample. Regarding marital status, most participants (95.6%) reported being single. In terms of academic progression, 29.3% of the participants were in their 5th year (4th year in medical school).

**Table 1:** Demographic characteristics of the studied medical students. *Includes different grade point average (GPA) systems, or students choosing 'I prefer not to answer'.

Characteristics	n	%
Gender		
Male	278	35.8
Female	499	64.2
Age		
<20	47	6.0
20-25	690	88.8
> 25	40	5.2
Marital status		
Single	743	95.6
Married	30	3.9
Divorced and widow	4	0.5
Monthly income		
Less than 5000 SAR	261	33.6
5000-<10000 SAR	97	12.5
10000-15000	96	12.4
>15000	323	41.6
Current year		
2nd year (First in medical school)	92	11.8
3rd year (2nd year in medical school)	109	14.0
4th year (3rd year in medical school)	182	23.4
5th year (4th year in medical school)	228	29.3
6th year (senior year in medical school)	166	21.4
Grade point average (GPA)		
4.5-5	356	45.8
3.75-4.4	213	27.4
2.75-3.74	57	7.3
<2.75	10	1.3
Others*	141	18.1

Table 2 illustrates the knowledge of medical students regarding plastic surgery. When asked if they had been exposed to the specialty, 25.4% responded affirmatively, whereas 74.6% answered negatively. Regarding the source of their plastic surgery knowledge, 26.7% cited lectures as their primary information channel. Furthermore, 42% of the participants acknowledged the availability of a plastic surgery residency program in their city, while 45.2% were unaware of it. Moreover, 52.3% expressed disinterest in undergoing a plastic surgery procedure themselves. A mere 10.9% indicated an extreme interest in pursuing plastic surgery as a specialty, with an even smaller proportion of the participants (6.1%) expressing an extreme interest and claiming familiarity with plastic surgery as a specialty.

**Table 2:** General knowledge of medical students regarding plastic surgery. *(1 = not at all interested, 5 = extremely interested)

	n	%
Have been previously exposed to plastic surgery?		
Yes	196	25.4
No	581	74.6
Source of exposure to plastic surgery		
Lectures	264	26.7%
Mandatory rotation	86	8.7%
Elective rotation	72	7.3%
Plastic surgeons faculty members	82	8.3%
No exposure	424	42.8%
Others	62	6.3%
Is there a plastic surgery residency program in your city?		
Yes	326	42.0
No	99	12.7
I don't know	352	45.3
Are you interested in undergoing/underwent a plastic surgery procedure on yourself?		
Yes	150	19.3
No	406	52.3
Maybe	221	28.4
How interested are you in pursuing Plastic Surgery as a specialty?*		
1.0	245	31.5
2.0	148	19.2
3.0	207	26.6
4.0	92	11.8
5.0	85	10.9
How knowledgeable are you about plastic surgery as a specialty?*		
1.0	177	22.8
2.0	230	29.6
3.0	235	30.2
4.0	87	11.2
5.0	48	6.1

Students’ preferences for surgical specialties in various clinical scenarios were also examined in this study (Tables 3-6). From Table 3, it is evident that 30% of the students opined that plastic surgery was the preferred specialty for repairing a finger severed in an accident. For scenarios involving the severing of the extensor tendon and carpal tunnel syndrome, 29.2% and 27.7% of the respondents, respectively, stated that orthopedics was the appropriate specialty. However, in cases of a knife cut to the ulnar nerve and brachial plexus injury, most respondents believed that neurology/neurosurgery was the suitable specialty. As presented in Table 4, 56% and 41.7% of the students indicated that orthopedics was the preferred specialty for repairing scaphoid bone fractures and jaw fractures, respectively. In contrast, for broken noses, 40.6% of the respondents cited plastic surgery as the suitable specialty. Regarding other conditions, such as repairing third-degree burns, burns covering 50% of the total body surface area, and addressing large pressure sores on the buttocks of patients with paraplegia, a significant proportion of the respondents, i.e., 47.5%, 48.4%, and 32.2%, respectively, identified plastic surgery to be the ideal specialty (Table 5). Furthermore, most students (57.1%) deemed plastic surgery suitable for repairing cleft lip anomalies. Also, for skull deformities affecting brain growth in a 2-year-old child and in children experiencing partial facial paralysis since birth, 31.1% and 38% of the respondents, respectively, opined that plastic surgery was the preferred specialty (Table 6).

**Table 3:** Students' choices of specialties for managing the provided scenarios - Part A. *Others included Ear, Nose, and Throat (ENT); Obstetrics; Urology; and other surgical-related departments.

Clinical Scenarios	n	%
1. Severed finger in an accident		
General surgery	399	26.7
Orthopedics	362	24.2
Plastic Surgery	435	30.0
Vascular Surgery	149	10.0
Others*	48	3.1
2. Severing the extensor tendon		
General surgery	265	23.2
Orthopedics	401	35.1
Plastic Surgery	334	29.2
Neurology/Neurosurgery	68	5.9
Vascular Surgery	55	4.8
Others*	21	1.8
3. Carpal tunnel syndrome		
Orthopedics	338	38.1
Plastic Surgery	245	27.7
Neurology/Neurosurgery	237	26.7
Vascular Surgery	42	4.7
Others*	24	2.5
4. Knife cut to the ulnar nerve		
General surgery	250	21.2
Orthopedics	191	16.2
Plastic Surgery	275	23.3
Neurology/Neurosurgery	354	30.0
Vascular Surgery	86	7.3%
Others	24	2.1
5. Brachial plexus injury		
General surgery	261	22.0%
Orthopedics	230	19.4%
Plastic Surgery	237	20.0%
Neurology/Neurosurgery	336	28.3%
Vascular Surgery	91	7.7%
Others*	29	2.7

**Table 4:** Students' choices of specialties for managing the provided scenarios - Part B. *Others included other surgical-related departments.

Clinical Scenarios	n	%
1. Scaphoid bone fracture		
General surgery	186	17.6%
Orthopedics	593	56.0%
Plastic Surgery	203	19.2%
Neurology/Neurosurgery	31	2.9%
Vascular Surgery	21	2.0%
Others*	8	0.8
2. Jaw fracture		
General surgery	136	11.3%
Orthopedics	502	41.7%
Plastic Surgery	398	33.1%
Ear, Nose, and Throat (ENT)	115	9.6%
Neurology/Neurosurgery	25	2.1%
Vascular Surgery	19	1.8
Others*	11	1.0
3. Broken nose		
General surgery	154	12.4%
Orthopedics	305	24.5%
Plastic Surgery	506	40.6%
Ear, Nose, and Throat (ENT)	236	18.9%
Neurology/Neurosurgery	12	1.0%
Vascular Surgery	19	1.5%
Others*	14	1.6

**Table 5:** Students' choices of specialties for managing the provided scenarios - Part C. *Others included other surgical-related departments.

Clinical Scenarios	n	%
1. Third degree burn		
General surgery	250	20.6%
Orthopedics	40	3.3%
Plastic Surgery	31	2.9%
Dermatology	228	18.8%
Neurology/Neurosurgery	48	3.9%
Others*	15	1.4
2. Burn covering 50% of total body surface area		
General surgery	222	18.0%
Orthopaedics	57	4.6%
Plastic Surgery	598	48.4%
Neurology/Neurosurgery	61	4.9%
Dermatology	210	17.0%
Vascular Surgery	66	5.3%
Others*	21	1.8
3. Paraplegic patient with large pressure sore at the buttock		
General surgery	303	27.0%
Orthopedics	90	8.0%
Plastic Surgery	361	32.2%
Neurology/Neurosurgery	186	16.6%
Dermatology	99	8.8%
Vascular Surgery	58	5.2%
Others*	25	2.4%

**Table 6:** Students' choices of specialties for managing the provided scenarios - Part D. *Others included other surgical-related departments.

Clinical Scenarios	n	%
1. Cleft lip anomaly		
General surgery	220	22.1%
Orthopedics	60	6.0%
Plastic Surgery	569	57.1%
Ear, Nose, and Throat (ENT)	114	11.4%
Neurology/Neurosurgery	14	1.4%
Dermatology	14	1.4%
Others*	6	0.7%
2. Skull deformity affecting brain growth for a 2 years old child		
General surgery	171	14.9%
Orthopedics	255	22.2%
Plastic Surgery	303	26.4%
Ear, Nose, and Throat (ENT)	26	2.3%
Neurology/Neurosurgery	357	31.1%
Vascular Surgery	19	1.7%
Others*	18	1.7%
3. Child with partial facial paralysis since birth		
General surgery	159	15.3%
Orthopedics	78	7.5%
Plastic Surgery	315	30.4%
Ear, Nose, and Throat (ENT)	36	3.5%
Neurology/Neurosurgery	402	38.8%
Obstetric and Gynecology	16	1.5%
Vascular Surgery	15	1.5
Others*	15	1.5%

Table 7 lists the factors that influence medical students’ decision to select plastic surgery as a specialty, categorized by sex. Compared with male students (36.9%), a higher percentage of female students (63.1%) reported that the internet was a key factor that influenced their impression of plastic surgery, although not significant. Moreover, compared with male students (32.3%), a greater proportion of female students (67.7%) watched medical dramas. The difference between the two sexes was statistically significant (P = 0.001). Female students (65.6%) also reported a higher percentage of personal encounter experiences influencing their impression of plastic surgery, compared with male students (34.4%). A similar pattern was observed for the influence of social media, where a higher proportion of female students (63.7%) cited it as a significant factor, compared with male students (36.3%), although not significant. Interestingly, a higher percentage of female students (69.3%) reported clinical rotations as a factor influencing their impression of plastic surgery, compared with male students (30.7%). In addition, a higher proportion of male students (61%) cited other factors, such as religion, books, journals, lectures, personal opinions, lack of interest, high income, and nonspecific factors, compared with female students (39%).

**Table 7:** Factors affecting choosing plastic surgery as a specialty by sex of students. *Other factors included religion, books, journals, lectures, personal opinion, not interested, high income.

	Gender		χ2	P-value	
	Male	Female			
Factors Influencing the Majority of Impressions on Plastic Surgery					
Internet	152 (36.9%)	260 (63.1%)	8.6		.37
Television Series	61 (35.7%)	110 (64.3%)			
Personal encounter experience	76 (34.4%)	145 (65.6%)			
Social media	163 (36.3%)	286 (63.7%)			
Clinical rotations	61 (30.7%)	138 (69.3%)			
Other factors*	8 (61%)	5 (39%)			
Do/did you watch any medical dramas?					
Yes	180 (32.3%)	377 (67.7%)	10.5		.001
No	98 (44.7%)	121 (55.3%)			
How often do you spend researching the topic online?					
Never	95 (38.6%)	151 (61.4%)	4.6		.20
Rarely	123 (37.4%)	206 (62.6%)			
Occasionally	49 (30.8%)	110 (69.2%)			
Often	11 (26.2%)	31 (73.8%)			

A comparison of these factors based on the year of study is presented in Table 8. As students progressed through their years of study, there appeared to be a trend toward increasing reliance on the internet, with higher proportions of students in later years (4th, 5th, and 6th years) citing it as an important influence, compared with those in earlier years. There was a notable increase in the influence of personal encounter experiences on students’ perceptions of plastic surgery as they progressed through their years of study, with the highest proportion reported by 6th-year students. However, according to the chi-square test, this trend was not statistically significant. The influence of clinical rotations on students’ perceptions of plastic surgery seemed to increase substantially as they progressed through their years of study, with the highest proportion reported by 6th-year students. Nonetheless, like other factors, this trend was not statistically significant.

**Table 8:** Factors affecting choosing plastic surgery as a specialty by the study year of the students. Other factors included religion, books, journals, lectures, personal opinion, not interested, high income.

	Year of Study					χ2	P-value
	2^nd^ year	3^rd^ year	4^th^ year	5^th^ year	6^th^ year		
Factors influencing the majority of impressions on plastic surgery							
Internet	52 (12.6%)	55 (13.3%)	99 (24.0%)	128 (31.1%)	78 (18.9%)	28.5	.101
Television Series	17 (9.9%)	22 (12.9%)	35 (20.5%)	55 (32.2%)	42 (24.6%)		
Personal encounter experience	15 (6.8%)	26 (11.8%)	51 (23.1%)	68 (30.8%)	61 (27.6%)		
Social media	52 (11.6%)	61 (13.6%)	107 (23.8%)	136 (30.3%)	93 (20.7%)		
Clinical rotations	14 (7.0%)	17 (8.5%)	36 (18.1%)	58 (29.1%)	74 (37.2%)		
Other factors*	2 (18.2%)	3 (27.2%)	4 (36.4%)	1 (9.1%)	1 (9.1%)		
Do/Did you watch any medical dramas?							
Yes	66 (11.8%)	79 (14.2%)	122 (21.9%)	172 (30.9%)	118 (21.2%)	3.8	.423
No	26 (11.9%)	30 (13.7%)	60 (27.4%)	55 (25.1%)	26 (11.9%)		
How often do you spend researching the topic online?							
Never	32 (13.0%)	28 (11.4%)	73 (29.7%)	66 (26.8%)	47 (19.1%)	18.4	.105
Rarely	34 (10.3%)	56 (17.0%)	73 (22.2%)	92 (28.0%)	74 (22.5%)		
Occasionally	18 (11.3%)	22 (13.8%)	30 (18.9%)	53 (33.3%)	36 (22.6%)		
Often	8 (19.0%)	3 (7.1%)	6 (14.3%)	16 (38.1%)	9 (21.4%)		

Table 9 presents the influence of various factors on medical students’ perceptions of plastic surgery, categorized by their GPA. Across all GPA categories, the internet was a key factor that influenced students’ impressions of plastic surgery, with relatively consistent proportions reporting it as influential. The influence of television series on students’ perceptions of plastic surgery was not significantly associated with GPA, as indicated by the chi-square test. The influence of social media on students’ perceptions of plastic surgery also did not exhibit a significant association with GPA. Likewise, GPA and the influence of personal encounter experience and clinical rotations on students’ perceptions of plastic surgery were not significantly associated. This could be attributed to the pervasive nature of these media forms, which may shape perceptions similarly across different academic performances. Students with both higher and lower GPAs reported watching medical dramas, but a significant association was lacking. Also, the frequency of researching the topic of plastic surgery online was not significantly associated with GPA, although this tendency was greater among higher-GPA students.

**Table 9:** Factors affecting choosing plastic surgery as a specialty by the grade point average (GPA) of the students. *Includes different grade point average (GPA) systems, or students choosing 'I prefer not to answer'. **Other factors include religion, books, journals, lectures, personal opinion, not interested, high income.

	GPA out of 5					χ2	P-value
	4.5 - 5	3.75 - 4.4	2.75 - 3.74	< 2.75	Others*		
Factors Influencing the Majority of Impressions on Plastic Surgery							
Internet	212 (51.5%)	102 (24.8%)	32 (7.8%)	3 (0.7%)	63 (15.3%)	27.5	.694
Television Series	70 (40.9%)	53 (31.0%)	13 (7.6%)	2 (1.2%)	33 (19.3%)		
Personal encounter experience	104 (47.1%)	61 (27.6%)	12 (5.4%)	2 (0.9%)	42 (19.0%)		
Social media	208 (46.3%)	120 (26.7%)	30 (6.7%)	3 (0.7%)	88 (19.6%)		
Clinical rotations	85 (42.7%)	58 (29.1%)	17 (8.5%)	2 (1.0%)	37 (18.6%)		
Other factors**	4 (40.0%)	3 (30.0%)	0 (0.0%)	0 (0.0%)	3 (30.0%)		
Do/did you watch any medical dramas?							
Yes	262 (47.0%)	152 (27.3%)	38 (6.8%)	5 (0.9%)	100 (18.0%)	3.8	.425
No	93 (42.5%)	61 (27.9%)	19 (8.7%)	5 (2.3%)	41 (18.7%)		
How often do you spend researching the topic online?							
Never	124 (50.4%)	70 (28.5%)	17 (6.9%)	3 (1.2%)	32 (13.0%)	12.4	.409
Rarely	142 (43.2%)	92 (28.0%)	23 (7.0%)	5 (1.5%)	67 (20.4%)		
Occasionally	73 (45.9%)	42 (26.4%)	11 (6.9%)	1 (0.6%)	32 (20.1%)		
Often	16 (38.1%)	9 (21.4%)	6 (14.3%)	1 (2.4%)	10 (23.8%)		

## Discussion

Plastic surgery, a dynamic field in medicine, is influenced by various factors that shape medical students’ perceptions and career choices. This study aimed to evaluate medical students’ perceptions of plastic surgery as a career and explore the demographic and educational factors affecting it. The study results indicated that only 25.4% of the participants were exposed to plastic surgery, whereas 74.6% were not. Students recognized plastic surgeons’ skills in trauma management, particularly for injuries like severed fingers. However, misconceptions exist about the role of plastic surgeons, as many students associated hand injuries with orthopedics or other specialties rather than plastic surgery. In terms of factors assessed, the data suggest that social media, medical dramas, and educational resources and engagement are highly influential in the choice of plastic surgery as a future career.

First, the relatively low exposure rate suggests a potential gap in the educational experiences of medical students regarding plastic surgery [7]. This lack of exposure could stem from various factors, such as the limited availability of plastic surgery rotations, insufficient emphasis on the specialty within the curriculum, and students’ personal choices in selecting clinical experiences [8]. Addressing this gap is crucial as exposure to different specialties during medical school aids students in making informed career decisions and fosters interest in various fields of medicine. Second, the disparity in exposure rates might have implications for the future workforce in plastic surgery. If a larger pool of students is exposed to the specialty, it could lead to more future plastic surgeons, and conversely, if a significant proportion of students do not have exposure, it could translate into fewer individuals considering plastic surgery as a career path, potentially leading to workforce shortages [9]. Moreover, insufficient exposure among medical students may hinder the availability of plastic surgery services in certain regions or result in delays in accessing care for patients in need of such interventions [9].

The findings regarding the sources of plastic surgery knowledge and awareness of residency programs among participants offer insights into the educational landscape and potential future trends in the field. The fact that 26.7% of the participants stated that lectures were the primary source of plastic surgery knowledge suggests that formal educational settings, such as lectures within the medical school curriculum, play a vital role in shaping students’ understanding of the specialty. Moreover, this observation highlights the importance of structured educational programs in providing foundational knowledge about plastic surgery to medical students [10]. However, this finding also indicates that a substantial proportion of students may rely on other sources or may not have access to comprehensive educational materials on plastic surgery.

Furthermore, the data pertaining to awareness of plastic surgery residency programs reveal notable trends. The fact that 42% of the participants acknowledged the availability of a plastic surgery residency program in their city shows a considerable level of awareness among the students. In a cross-sectional study that included 507 medical students, Mortada et al. [4] reported a high mean score of awareness about plastic surgery, particularly among female students. This awareness is crucial as it can influence students’ career decisions, especially if they get opportunities for hands-on experiences or mentorship within these residency programs. In contrast, the fact that 45.2% of the participants were unaware of the presence of such programs signifies potential gaps in communication or outreach efforts regarding plastic surgery training opportunities. This lack of awareness may limit students’ ability to consider plastic surgery as a career option or pursue relevant training opportunities, ultimately affecting the future workforce in this specialty [2,10].

The finding that only a small percentage of students expressed extreme interest in pursuing plastic surgery as a specialty (10.9%), with an even smaller fraction claiming familiarity with the field (6.1%), raises important considerations about the factors influencing medical students’ career choices and perceptions of plastic surgery. A recent cross-sectional study involving 244 medical students at Kuwait University found that only 15.5% were interested in enrolling in a plastic surgery residency after graduation, and that 47.1% did not consider it [11]. These observations suggest that plastic surgery is not widely perceived as a highly desirable career option among medical students [10,11]. Nevertheless, other studies have reported a much higher proportion of students preferring plastic surgery as a specialty career. For instance, in one study, 53.1% of the students stated that they would choose plastic surgery as their future specialty [12], and in another study, 25% of the participants [13] rated it as the most alluring specialty.

The findings from this study indicate that 30% of the students preferred plastic surgery for repairing a severed finger, which shows that they recognize plastic surgeons’ proficiency in managing traumatic injuries and performing intricate reconstructive procedures. Moreover, this outcome reflects students’ understanding of the dual role of plastic surgery in addressing both functional and aesthetic concerns related to hand injuries. In contrast, another study reported that 79.8% of medical students perceived plastic surgery as crucial in trauma management and that 9.2% did not consider it appropriate for this purpose [11]. In the present study, for scenarios involving the severing of the extensor tendon and carpal tunnel syndrome, 29.2% and 27.7% of the students identified orthopedics as the appropriate specialty, respectively. However, for cases of a knife cut to the ulnar nerve and brachial plexus injury, most respondents opined that neurology/neurosurgery was the suitable specialty. Another similar study documented that over half of the students believed that hand surgeons were not plastic surgeons [8]. This finding may be explained by several factors, such as the decreasing number of plastic surgeons performing hand surgery, the presence of an orthopedic designated hand fellowship in several medical colleges, and limited training exposure, with only 30% of the students being exposed to plastic surgery during their four years of training [8].

When the preferences of medical students regarding the specialties appropriate for bone fracture scenarios were questioned, orthopedics emerged as the favored specialty for repairing scaphoid bone fractures and jaw fractures, with 56% and 41.7% of the respondents opting for it, respectively. Conversely, for broken noses, 40.6% of the students identified plastic surgery as the suitable specialty. This finding agrees with the results of recent studies examining such scenarios among medical students [13,14]. When students’ perceptions regarding their treatment preferences for burns and bed sores were queried, almost half of the respondents felt that plastic surgery was the optimal choice for treating third-degree burns. Furthermore, nearly one-third of the students found plastic surgery to be appropriate for managing large pressure sores on the buttocks of patients with paraplegia. Similarly, more than half of the students (57.1%) considered it suitable for repairing cleft lip anomalies. However, for more complex conditions, such as skull deformities affecting brain growth in young children and partial facial paralysis since birth, a smaller but notable percentage of students (31.1% and 38%, respectively) identified plastic surgery as the preferred specialty. Collectively, these findings signify that while orthopedics remains a popular choice for certain bone-related injuries, plastic surgery is increasingly recognized as a versatile specialty to address a wide range of conditions, including burns, pressure sores, and congenital anomalies.

The findings pertaining to the factors that influence students’ perceptions of plastic surgery emphasize the key role of media in shaping attitudes and beliefs about medical specialties. The fact that nearly one-third of the students cited social media as the primary influencer highlights the profound impact of online platforms on contemporary society. Similar regional and international studies have reported that social media platforms provide a wealth of information, including patient testimonials, before-and-after photos, and educational content about plastic surgery [2,4,10-14]. However, the accuracy and reliability of information on social media vary, potentially leading to misconceptions or unrealistic expectations among students [14]. Likewise, the influence of the internet, reported by 28.1% of the students, showcases the accessibility of information online and the ease with which students can obtain resources related to plastic surgery. Although the internet offers valuable educational resources, there are challenges in distinguishing credible sources from misinformation, especially in the realm of healthcare [15].

Furthermore, this study revealed that most students (71.7%) cited medical dramas as an influential factor in their career choice, underscoring the impact of popular culture in shaping students’ perceptions of medical specialties. Other studies have also observed that medical dramas are an important influencing factor in the selection of plastic surgery as a career [11,16]. In addition, television and other media should be encouraged to provide comprehensive information about the true role of plastic surgeons to the public, including medical students [17]. This finding highlights the importance of critically evaluating media representations of healthcare professions and knowing the difference between fictional portrayals and real-world practice.

Additionally, the finding that 42.3% of the students admitted to rarely researching the topic online suggests a potential gap in proactive engagement with educational resources about plastic surgery. While social media and medical dramas can offer exposure to the field, active online research and inquiry are imperative to develop a comprehensive understanding of the specialty and make informed career decisions [18].

When examining the influencing factors based on students’ demographic and educational characteristics, no statistically significant differences were found. However, regarding the influence of watching medical dramas, 67.7% of female students admitted to being influenced by it, significantly differing from male students. The fact that watching medical dramas significantly influenced female students more than their male counterparts indicates potential differences in how the two sexes are impacted by media representations of healthcare professions. A recent systematic review concluded that female medical students were positively influenced by mentorship, intellectual challenge, the rewarding nature of surgery, and specialty exposure. Gender discrimination, surgical lifestyle, and societal and cultural barriers were the key deterrents for female medical students [19].

Strengths and limitations 

This study has provided detailed demographic information about the participants, including sex, age, marital status, academic level, and university affiliation. This comprehensive characterization of the study sample has enhanced the understanding of the population under investigation and has facilitated the interpretation of findings. Furthermore, this study has assessed medical students’ exposure to and knowledge of plastic surgery, as well as their preferences for surgical specialties in various clinical scenarios. Thus, this study has offered beneficial insights into the factors that influence the career choices of medical students. These findings can inform medical education and career counseling programs. Unlike other regional studies, participants from multiple universities were included in this study, thereby providing a broad representation of medical students across Saudi Arabia. This multicenter approach has augmented the generalizability of the findings beyond a single institution or region.

However, despite these strengths, this study might have been subjected to sampling bias due to the use of convenience sampling methods. For example, students from urban universities may have been overrepresented, while those from rural areas might have been underrepresented, potentially skewing the results. This limitation could affect the generalizability of the findings to the broader population of medical students in Saudi Arabia, particularly if the sample does not accurately reflect the diversity of the overall student population.

Moreover, the study relied on self-reported data, which is susceptible to recall bias or social desirability bias. Participants might have provided responses they believed to be socially acceptable rather than reflecting their true experiences or opinions, which could affect the validity of the findings. To mitigate these biases, future studies could employ mixed methods, such as combining quantitative surveys with qualitative interviews, allowing for a more nuanced understanding of participants' perspectives. Additionally, triangulation of data sources can provide a more comprehensive view and help cross-verify findings.

Specific steps taken to enhance the representativeness of the sample included outreach to various universities and ensuring diverse participant recruitment. However, the limitations related to convenience sampling and self-reported data must be acknowledged, as they could impact the interpretation of the findings. For instance, if certain demographic groups were less likely to participate, the insights gained might not fully capture the career aspirations of all medical students in the region.

## Conclusions

This study has provided valuable insights into the factors influencing the perceptions of medical students in Saudi Arabia regarding plastic surgery as a career option. The findings have emphasized the multifaceted nature of factors influencing students' perceptions, which range from exposure to educational experiences and media influences. Despite the modest overall interest in plastic surgery, the observations from this investigation highlight the importance of early exposure, targeted educational interventions, and mentorship initiatives in cultivating interest and dispelling misconceptions about this specialty. Moreover, the differences in the manner in which the two sexes are influenced by media representations highlight the need for tailored approaches to managing students from diverse demographic backgrounds in medical education. Early interventions to correct misconceptions and enhance exposure to this specialty during medical training are crucial for cultivating keen interest and developing a knowledgeable workforce in plastic surgery.

Future studies are needed to explore possible curriculum changes and initiatives based on the current perception findings and the common misconceptions, and to measure their impact on medical students.

## References

[1] Rohrich RJ, McGrath MH, Lawrence WT, Ahmad J (2010). Assessing the plastic surgery workforce: a template for the future of plastic surgery. Plast Reconstr Surg.

[2] Fraser SJ, Al Youha S, Rasmussen PJ, Williams JG (2017). Medical student perception of plastic surgery and the impact of mainstream media. Plast Surg (Oakv).

[3] Alyahya T, Zakaria OM, Al Jabr FA (2021). Plastic and aesthetic surgery among medical students: a cross-sectional study. SAGE Open Med.

[4] Mortada HH, Alqahtani YA, Seraj HZ, Albishi WK, Aljaaly HA (2019). Perception of plastic surgery and the role of media among medical students: cross-sectional study. Interact J Med Res.

[5] Morzycki A, Bezuhly M, Williams JG (2018). How competitive is plastic surgery? An analysis of the Canadian and American residency match. Plast Surg (Oakv).

[6] Aljindan F, Zahrani G, Almalki N (2021). Knowledge and perception of plastic surgery among medical students in the Western Region of Saudi Arabia. J Healthc Sci.

[7] Kling RE, Nayar HS, Harhay MO, Emelife PO, Manders EK, Ahuja NK, Losee JE (2014). The scope of plastic surgery according to 2434 allopathic medical students in the United States. Plast Reconstr Surg.

[8] Agarwal JP, Mendenhall SD, Moran LA, Hopkins PN (2013). Medical student perceptions of the scope of plastic and reconstructive surgery. Ann Plast Surg.

[9] Mendenhall S, Agarwal J (2013). Improving medical student understanding of the scope of plastic surgery. Ann Plast Surg.

[10] Denadai R, Raposo-Amaral C (2014). Undergraduate plastic surgery education: problems, challenges, and proposals. Ann Med Health Sci Res.

[11] Abdulaziz MK, Al-Jamali M, Al-Mazidi S, Albuloushi S, Al-Ali AB (2024). Medical students understanding of the scope of plastic surgery. Arch Plast Surg.

[12] Al Qurashi AA, Shah Mardan QN, Mortada H, Maddawi H, Hakami AY, Mrad MA (2021). Factors influencing the choice of plastic surgery as a specialty in Saudi Arabia. Plast Reconstr Surg Glob Open.

[13] Pasha T, Lumley ES, Dwyer-Hemmings L, Fell M (2020). Undergraduate plastic surgery in the United Kingdom: the students' perspective. J Plast Reconstr Aesthet Surg.

[14] Rogers AD, dos Passos G, Hudson DA (2013). The scope of plastic surgery. S Afr J Surg.

[15] Alshahrani M, Dhafery B, Al Mulhim M, Alkhadra F, Al Bagshi D, Bukhamsin N (2014). Factors influencing Saudi medical students and interns' choice of future specialty: a self-administered questionnaire. Adv Med Educ Pract.

[16] Asiri WM, Shati AA, Alrowaibah NA, Althumairi RK, Alqahtani GM, Mahmood SE (2023). The influencing factors of choosing future medical specialties among students in Saudi Arabia: a nationwide multicenter survey. Medicine (Baltimore).

[17] Reid AJ, Malone PS (2008). Plastic surgery in the press. J Plast Reconstr Aesthet Surg.

[18] Shauly O, Marxen T, Goel P, Gould DJ (2023). The new era of marketing in plastic surgery: A systematic review and algorithm of social media and digital marketing. Aesthet Surg J Open Forum.

[19] Trinh LN, O'Rorke E, Mulcahey MK (2021). Factors influencing female medical students’ decision to pursue surgical specialties: a systematic review. J Surg Educ.

